# (-)-*cis*-Carveol, a Natural Compound, Improves *β*-Amyloid-Peptide 1-42-Induced Memory Impairment and Oxidative Stress in the Rat Hippocampus

**DOI:** 10.1155/2020/8082560

**Published:** 2020-04-22

**Authors:** Lucian Hritcu, Razvan Stefan Boiangiu, Mayara Castro de Morais, Damião Pergentino de Sousa

**Affiliations:** ^1^Department of Biology, Faculty of Biology, Alexandru Ioan Cuza University of Iasi, Bd. Carol I, No. 11, 700505 Iasi, Romania; ^2^Department of Pharmaceutical Sciences, Federal University of Paraíba, 58051-970 João Pessoa, PB, Brazil

## Abstract

Alzheimer's disease (AD) could be considered a multifactorial neurodegenerative disorder characterized by the accumulation of the *β*-amyloid-peptide (A*β*) within the brain leading to cognitive deficits, oxidative stress, and neuroinflammation. The present work was carried out to investigate the neuroprotective effect of (-)-*cis*-carveol (1% and 3%, for 21 days) against the *β*-amyloid-peptide 1-42- (A*β*1-42-) induced AD. Twenty-five rats were divided into five groups (*n* = 5/group): the first group—control (sham-operated); the second group—A*β*1-42 (1 mM) that received donepezil treatment (5 mg/kg, as the positive reference drug in the Y-maze and the radial arm maze tests); the third group—A*β*1-42 (1 mM); the fourth and fifth groups—A*β*1-42 (1 mM) that received (-)-*cis*-carveol treatment groups (1% and 3%). The results of this study demonstrated that (-)-*cis*-carveol improved A*β*1-42-induced memory deficits examined by using Y-maze and radial arm maze *in vivo* tests. Also, the biochemical analyses of the hippocampus homogenates showed that (-)-*cis*-carveol reduced hippocampal oxidative stress caused by A*β*1-42. Our results suggested that the use of (-)-*cis*-carveol may be suitable for decreasing AD-related symptoms.

## 1. Introduction

Alzheimer's disease (AD) is considered an irreversible, progressive neurological disorder characterized by the accumulation of the extracellular *β*-amyloid-peptide (A*β*), intracellular neurofibrillary tangles, disruption of synapses, brain inflammation, and memory loss [1]. Also, there is some evidence that neuropsychiatric symptoms, particularly anxiety and depression, could be risk factors for cognitive impairment and AD dementia and are also associated with cortical A*β* deposition [2, 3]. Furthermore, there is evidence between oxidative stress and neuronal dysfunction in AD [4]. Also, excessive A*β* may trigger stress-related signaling pathways through increasing Ca^2+^ influx, increasing oxidative stress, and impairing energy metabolism [4, 5]. At present, clinical drug treatments are limited to acetylcholinesterase inhibitors (AChEIs), such as donepezil and the antagonist of N-methyl-D-aspartic acid (NMDA) receptor, represented by memantine [6]. Unfortunately, recent failures and limited progress of therapeutics suggest that alternative strategies for AD treatment could be considered [7].

Essential oils are natural products that demonstrate high therapeutic potential against various types of pathologies, including central nervous system (CNS) disorders. Their chemical constituents exhibit various pharmacological activities such as anxiolytic [8], antidepressant [9], analgesic [10], anticonvulsant [11], and inhibitory effects against brain tumor cells [12]. Monoterpenes are compounds commonly found in essential oils that have received much attention as a potential therapy for AD [13]. It has been documented that carvacrol, a monoterpenoid phenol, significantly reduced the development of cerebral edema [14]. Linalool, terpene alcohol, improved learning and memory in a transgenic mouse model of AD by reduction of the level of inflammation markers [15]. Khazdair et al. [16] reported that monoterpenes from various medicinal plants are active constituents with therapeutic potential in disorders associated with neuroinflammation and neurotransmitter deficiency such as AD and depression.

Carveol is monocyclic monoterpenoid alcohol with a *p*-menthane skeleton found in essential oils of plants, such as *Cymbopogon giganteus* [17], *Illicium pachyphyllum* [18], and *Carum carvi* [19]. Its chemical structure is similar to several neuro- and psychoactive terpenes [8, 11], such as (-)-perillyl alcohol, which has been demonstrated to take antinociceptive action [20], in addition to anticancer activity against brain tumor [21]. Therefore, in the present investigation, we aimed to assess if the enantiomer (-)-*cis*-carveol could improve the *β*-amyloid-peptide 1-42- (A*β*1-42-) induced memory impairment and oxidative stress in the rat hippocampus.

## 2. Materials and Methods

### 2.1. Chemical Characterization and Reagents

The ^1^H- and ^13^C-NMR measurements were obtained with a Mercury-Varian spectrometer operating at 200 MHz for ^1^H, and 50 MHz for ^13^C. The infrared spectra were recorded on a Bomen Michelson model 102 FTIR and the bands reported in cm^−1^. Optical rotations were measured on an Optical Activity AA-10 automatic polarimeter at ambient temperature. For the isolation and purification of the products, column adsorption chromatography (silica gel 60, ART 7734, MERCK, St. Louis, Missouri, EUA) on hexane and ethyl acetate (EtOAc) gradients was used. Reaction monitoring and product analysis were via thin layer analytical chromatography (silica gel 60 F254) and ultraviolet light visualization using two wavelengths (254 and 366 nm). Deuterated solvent CDCl_3_ (CAS 865-49-6) was used. Chemical shifts (d) were measured in parts per million (ppm) with coupling constants (*J*) in Hz. The reagents and solvents used for the preparation of the compounds were of technical quality or P.A. (-)-Carvone (CAS 6485-40-1), cesium chloride heptahydrate (CeCl_3_.7H_2_O) (CAS 18618-55-8), diethyl ether (CAS 60-29-7), and sodium borohydride (CAS 16940-66-2) were purchased from Sigma-Aldrich. Methanol (MeOH) (CAS 67-56-1), hexane (CAS 110-54-3), and EtOAc (CAS 141-78-6) were obtained from the company Vetec (Duque de Caxias, Brazil).

### 2.2. Preparation of the Monoterpene (-)-*cis*-Carveol

The monoterpene (-)-*cis*-carveol was obtained through the reduction reaction on the enantiomer (-)-carvone and using sodium borohydride as a reducing agent, in the presence of cesium chloride heptahydrate, as previously published [22, 23].

### 2.3. Procedure for Reduction of (-)-Carvone

Sodium borohydride (2.5 g, 66.1 mmol) was added to (-)-carvone (10 g, 67 mmol) and CeCl_3_.7H_2_O (25 g, 148.5 mmol) in MeOH (500 mL) at 23°C. The solution was stirred for 5 min. Then, diethyl ether (100 mL) and water (100 mL) were added. The organic layer was separated, and the aqueous layer was extracted with diethyl ether (3 × 100 mL). The organic phase was dried over anhydrous sodium sulfate, and the solvent was evaporated under reduced pressure. The (-)-*cis*-carveol was isolated on a silica gel 60 chromatographic column using hexane and EtOAc (8 : 2) as eluent [22, 23].

### 2.4. Animals and Drug Administration

**Figure 1:**
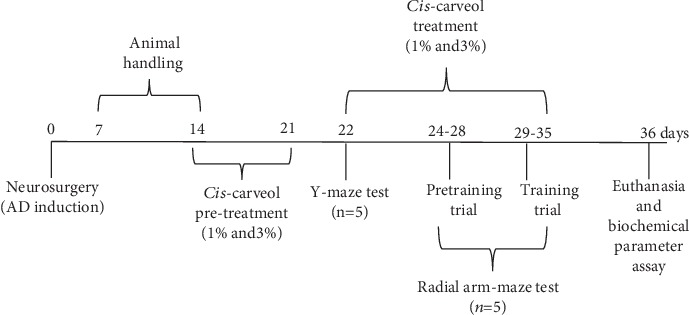
Experimental design.

Twenty-five adult male Wistar rats were used in this study (300 ± 50 g; purchased from Cantacuzino Institute, Bucharest, Romania). The animals were kept in a temperature and light-controlled room (22°C, a 12 h cycle starting at 08:00 h) with free access to food and water. Rats were divided into five groups (*n* = 5/group): the first group—control (sham-operated); the second group—A*β*1-42 (1 mM) that received donepezil treatment (5 mg/kg, as the positive reference drug in the Y-maze and the radial arm maze tests); the third group—A*β*1-42 (1 mM); the fourth and fifth groups—A*β*1-42 (1 mM) received (-)-*cis*-carveol treatment groups (1% and 3%). The control, A*β*1-42+donepezil, and A*β*1-42 groups received 1% Tween 80 solution through inhalation. (-)-*cis*-Carveol was diluted with 1% Tween 80 (*v*/*v*), and exposure (200 *μ*L, either 1% or 3%) was via an electronic vaporizer (KBAYBO). Regarding concentrations to be used in the pharmacological tests, we chose the dose of 1% for the carveol in the same way as it is used for the essential oil in aromatherapy and a higher dose (3%) to emphasize the effects [24]. Rats were pretreated by inhalation with (-)-*cis*-carveol (1% and 3%) for seven days before starting behavioral tests and continuously administered during behavior tests (21 days). Donepezil hydrochloride (Sigma-Aldrich, Germany) was dissolved in 0.9% physiological saline (5 mg/kg) and injected i.p., once daily, 30 min before the Y-maze and radial arm maze tasks. Also, we confirm that *n* = 5 animals/group is appropriate using InVivoStat and R-based statistical package [25]. Based on a significance level of 0.05, the power to detect a 20% biologically relevant change from control is 99%. All experimental procedures were strictly conducted by the Directive 2010/63/EU of the European Parliament and of the Council of 22 September 2010 on the protection of animals. The experimental process is indicated in Figure 1. Also, for the behavioral and biochemical parameter assays, we followed the methods of Postu et al. [26].

### 2.5. Neurosurgery

Under sodium pentobarbital (50 mg/kg b.w., i.p., Sigma-Aldrich, Germany) anesthesia, 1 mM aggregated A*β*1-42 in sterile saline solution (Sigma-Aldrich, Germany) was intracerebroventricularly (i.c.v.) delivered to rats on day 0 as discussed by Postu et al. [26]. The infusion volume (4 *μ*L) was injected gradually (1 *μ*L/min) utilizing the following coordinates: 1.5 mm lateral to the midline and 7.4 mm ventral to the surface of the cortex [27]. The control group (sham-operated animals) got an identical volume of the saline solution rather than the A*β*1-42 solution. The behavioral tests were done from the 22^nd^ day (Y-maze) and the 24^th^ day (radial arm maze) after neurosurgery (Figure 1) and were performed blind to the treatments by the observer.

### 2.6. Behavioral Assays

#### 2.6.1. Y-Maze Test

The impact of (-)-*cis*-carveol (1% and 3%) on the spontaneous alternation behavior was confirmed in a single-session Y-maze on the 22^th^ day postsurgery [26, 28]. The Y-maze used in the present study was constructed of Plexiglas having the following dimensions: 25 cm high, 35 cm long, and 10 cm wide of each arm and an equilateral triangular area. 15 min after receiving (-)-*cis*-carveol (1% and 3%) by inhalation, each animal was put at the end of one arm and permitted to move openly for an 8 min session. Spontaneous alternation behavior was characterized as consecutive entry into all three arms on covering triplet sets. The spontaneous alternation percentage (SAP) was calculated as follows: SAP (%) = (number of alternation/number of total arm entries − 2) × 100. The animal behavior was recorded using a Logitech HD Webcam C922 Pro Stream camera, and the videos were analyzed with ANY-maze® software (Stoelting CO, USA). The Y-maze was washed with a 10% ethanol solution in trials.

#### 2.6.2. Radial Arm Maze Test

The impacts of (-)-*cis*-carveol (1% and 3%) on the spatial memory were tested by employing a radial arm maze for 7 days, starting with the 24^th^ day postsurgery [26, 29]. The maze, which consisted of eight arms numbered from 1 to 8 (48 cm × 12 cm), with a radial extension of 32 cm in diameter from the central field, had 50 mg of food pellets at the end of arms 1, 2, 4, 5, and 7. Four days of sessions on habituation were conducted. Rats have been instructed to move to the end of the arms and eat the pellet of food during 5 min sessions. After habituation, all rats were given just one trial per day. For working and reference memory assignments, each rat was separately put within the center of the maze, taking 15 min administration by inhalation of (-)-*cis*-carveol (1% and 3%). Measures were made by (i) evaluating the number of working memory errors (entering an arm containing food, but previously entered) and (ii) assessing the reference memory errors by checking the animal enters in an arm without bait. A Logitech HD Webcam C922 Pro Stream camera recorded the animal behavior, and the ANY-maze® software (Stoelting CO, USA) was used for the video's analyses. The radial arm maze was washed with a 10% ethanol solution in trials.

### 2.7. Biochemical Assay

The hippocampal tissue samples were individually homogenized and finally centrifuged (15 min at 960 × *g*). The resulting supernatants had been used for the determination of SOD-, CAT-, and GPX-specific and AChE activities, along with reduced GSH, protein carbonyl, and MDA levels.

#### 2.7.1. Determination of the Hippocampal AChE Activity

For the evaluation of acetylcholinesterase (AChE) activity, an earlier described method used by Ellman et al. [30] was used. The final volume of the reaction mixture (600 *μ*L) contained 0.26 M phosphate buffer with pH 7.4, 1 mM 5.5′-dithio-bis-2 nitrobenzoic acid (DTNB), and 5 mM acetylthiocholine chloride (ATC). The assay was started by a adding supernatant and then following the development of the yellow color at room temperature at 412 nm for 10 min. Suitable controls for ATC's nonenzymatic hydrolysis were performed. The enzyme activity was formulated as nmol of ACT/min per/mg of protein.

#### 2.7.2. Determination of the Hippocampal SOD Activity

For the determination of the activity of superoxide dismutase (SOD, EC 1.15.1.1), the method described previously by Winterbourn et al. [31] was applied. There were 100 mM TRIS/HCl (pH 7.8), 75 mM NBT, 2 *μ*M riboflavin, 6 mM EDTA, and 200 *μ*L supernatant in each 1.5 mL reaction mixture. The monitoring of the absorbance increases at 560 nm following blue formazan output. One unit of SOD is classified as the amount needed to inhibit the nitroblue etrazolium (NBT) reduction rate by 50%. The enzyme activity was reported in units/mg protein.

#### 2.7.3. Determination of the Hippocampal CAT Activity

For the evaluation of the catalase (CAT, EC 1.11.1.6) activity, a previously used method described by Sinha [32] was applied. The reaction mixture was composed of 150 *μ*L phosphate buffer (0.01 M, pH 7.0) and 100 *μ*L supernatant. The reaction was initiated by adding 250 *μ*L H_2_O_2_ 0.16 M, incubated at 37°C for 1 min, and then the reaction was stopped by the addition of 1 ml of dichromate: acetic acid reagent. The tubes were immediately kept in a boiling water bath for 15 min, and the green color formed during the reaction was read at 570 nm by using a spectrophotometer. Control tubes, devoid of the enzyme, were also processed in parallel. The activity of the enzyme is expressed as *μ*mol of H_2_O_2_ consumed/min/mg protein.

#### 2.7.4. Determination of the Hippocampal GPX Activity

For the assessment of the glutathione peroxidase (GPX, E.C. 1.11.1.9) activity, an approach previously described by Sharma and Gupta [33] was used. A reaction mixture consisting of 1 mL 0.4 mM phosphate buffer (pH 7.0) containing 0.4 mM EDTA, 1 mL of 5 mM NaN_3_, 1 mL of 4 mM glutathione (GSH), and 200 *μ*L of supernatant was preincubated at 37°C for 5 min. Then, 1 mL of 4 mM H_2_O_2_ was inserted and incubated for another 5 min at 37°C. The GSH excess was quantified using the DTNB method. One unit of GPX is specified as the amount of enzyme needed to oxidize for 1 nmol GSH/min. The enzyme activity was expressed as units/mg protein.

#### 2.7.5. The Total Hippocampal Content of Reduced GSH

For the measurement of the reduced glutathione (GSH) content, the method of Fukuzawa and Tokumura [34] was used. A 200 *μ*L supernatant was applied to 1.1 mL of 0.25 M sodium phosphate buffer (pH 7.4), followed by the introduction of 130 *μ*L DTNB 0.04%. Finally, the mixture was taken to a final volume of 1.5 mL with distilled water, and the absorbance was read at 412 nm using a spectrophotometer. Results were shown as *μ*g GSH/*μ*g protein.

#### 2.7.6. Determination of Hippocampal Protein Carbonyl Level

The extent of protein oxidation in the hippocampus was assessed by measuring the content of protein carbonyl groups, using a method described by Oliver et al. [35] and modified through Luo and Wehr [36]. The supernatant fraction was split into two equal aliquots each that contained around 2 mg of protein. Both aliquots were precipitated using 10% trichloroacetic acid (TCA (*w*/*v*), final concentration). Another sample was treated with 2 N HCl, and another sample was treated with 0.2% (*w*/*v*) DNPH in 2 N HCl at equivalent volume. Both samples were incubated at 25°C and then stirred at intervals of 5 min. The results had been expressed as nmol/mg protein.

#### 2.7.7. Determination of Hippocampal MDA Level

The content of malondialdehyde (MDA), an indicator of lipid peroxidation, was measured via the usage of the approach previously described [37]. 200 *μ*L of supernatant was applied and briefly mixed in 0.1 M HCl with 1 mL of 50% TCA in 0.1 M HCl and 1 mL of 26 mM thiobarbituric acid. Samples were held at 95°C for 20 min after vortex mixing. Samples were then centrifuged for 10 min at 960 × *g*, and the supernatants were read at 532 nm. The findings were presented as nmol/mg protein, as stated.

#### 2.7.8. Estimation of Protein Concentration

The evaluation of protein was accomplished by a bicinchoninic acid (BCA) protein assay kit from Sigma-Aldrich, Germany, following an approach described by Smith et al. [38].

### 2.8. Statistical Analysis

Data are expressed as the means ± SEM. The statistical evaluation was done through a one-way analysis of variance (ANOVA) accompanied by Tukey's *post hoc* test. Results were analyzed with GraphPad Prism 7 software, and the values of *F* for which *p* < 0.05 had been considered to indicate statistical significance.

## 3. Results and Discussion

(-)-*cis*-Carveol was obtained with a high yield (80%; 53.46 mmol). Analytical and spectroscopic data and comparison with literature data confirmed the chemical structure of this monoterpene [22, 23].

### 3.1. Structural Characterization of (-)-*cis*-Carveol

[*α*]_*D*_^29^ = −33.7^o^(CHCl_3_, *c* 0.03); IR (KBr) *ν*_max_: 3461, 2945, 2900, 1650, 1500, 1050, 900 cm^–1^; ^1^H-NMR (CDCl_3_): *δ* 5.44–5.39 (1H, m), 4.93 (2H, s), 4.25–4.21 (1H, dd, *J* = 8 *Hz*), 2.42–2.26 (5H, m), 2.22–2.12 (3H, m), 1.65 (3H, s), 1.56 (1H, s); ^13^C-NMR (CDCl_3_) *δ*: 146.3, 134.2, 125.3, 106.5, 68.7, 38.3, 36.7, 28.2, 27.2, 26.5 [23].

### 3.2. Effects of (-)-*cis*-Carveol on Spatial Memory Impairment in Y-Maze and Radial Arm Maze Tests Induced by A*β*1-42

**Figure 2:**
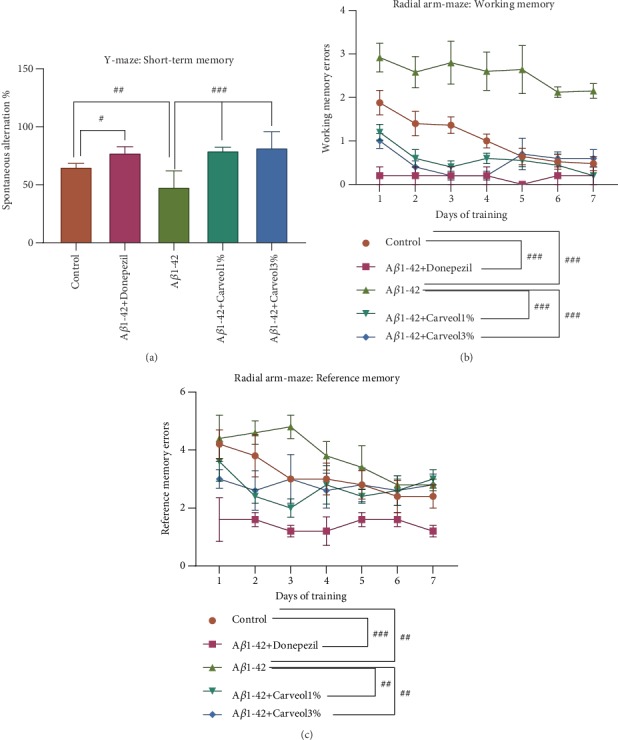
Effects of the inhaled carveol (1% and 3%) on (a) the SAP (%) in the Y-maze task and on (b) the working memory errors and (c) the reference memory errors during 7 days training in the radial arm maze task in the A*β*1-42-treated rats. Values are the means ± S.E.M. (*n* = 5 animals per group). For Tukey's *post hoc* analyses: (a) control vs. A*β*1-42+Donepezil—^#^*p* < 0.01, control vs. A*β*1-42—^##^*p* < 0.001, A*β*1-42 vs. A*β*1-42+Carveol1%—^###^*p* < 0.0001, and A*β*1-42 vs. A*β*1-42+Carveol3%—^###^*p* < 0.0001; (b) control vs. A*β*1-42+Donepezil—^###^*p* < 0.0001, control vs. A*β*1-42—^###^*p* < 0.0001, A*β*1-42 vs. A*β*1-42+Carveol1%—^###^*p* < 0.0001, and A*β*1-42 vs. A*β*1-42+Carveol3%—^###^*p* < 0.0001; (c) control vs. A*β*1-42+Donepezil—^###^*p* < 0.0001, control vs. A*β*1-42—^##^*p* < 0.001, A*β*1-42 vs. A*β*1-42+Carveol1%—^##^*p* < 0.001, and A*β*1-42 vs. A*β*1-42+Carveol3%—^##^*p* < 0.001.

In the Y-maze test, one-way ANOVA revealed overall significant differences between groups (*F*(4, 20) = 11.70, *p* < 0.0001) (Figure 2(a)). The data showed a substantial decline of the SAP (%) in the A*β*1-42 group as compared to the control group (*p* < 0.001), indicating that the A*β*1-42 rats had memory impairment following A*β*1-42 injection. Consequently, the (-)-*cis*-carveol-treated groups and the donepezil-treated group (*p* < 0.01) significantly attenuated the decreasing of SAP in the A*β*1-42 injected rats (*p* < 0.0001).

One-way ANOVA showed significant overall differences between groups for working memory errors (*F*(4, 20) = 54.49, *p* < 0.0001) (Figure 2(b)) and for reference memory errors (*F*(4, 20) = 17.29, *p* < 0.0001) (Figure 2(c)). Compared with the control group, the A*β*1-42 group displayed a significant number of working memory errors (*p* < 0.0001) which were decreased by inhalation of the (-)-*cis*-carveol (*p* < 0.0001 for (-)-*cis*-carveol 1% and *p* < 0.0001 for (-)-*cis*-carveol 3%). Also, donepezil showed significant effects on those of (-)-*cis*-carveol treatment on working memory errors (*p* < 0.0001). On the other hand, the A*β*1-42 group performed a considerable number of reference memory errors as compared to the control group (*p* < 0.001), which were reduced by the (-)-*cis*-carveol treatment (*p* < 0.001 for (-)-*cis*-carveol 1% and *p* < 0.001 for (-)-*cis*-carveol 3%). Donepezil administration significantly reduced the reference memory errors (*p* < 0.0001). Behavioral experiments showed that inhalation of (-)-*cis*-carveol improved the spatial memory in an A*β*1-42-induced rat model of AD.

Our results are in line with previous studies where chronic treatment with carvacrol, a phenolic monoterpene abundantly presented in the essential oil of the Lamiaceae family, improved memory deficits in the 6-hydroxydopamine Parkinson's disease (PD) rat model [39]. Supporting evidence suggested many pharmacological effects of the carvacrol, including antibacterial, antifungal, antioxidant, antinociceptive, anti-inflammatory, antiapoptosis, and anticancer activities [40]. Also, carvacrol exhibited AChE inhibition [41] as well as having anxiolytic [42] and antidepressant properties [43]. Moreover, our group demonstrated that monoterpene-rich essential oil from *Pinus halepensis* improved memory function in an A*β*1-42 rat model [26]. The present study showed that (-)-*cis*-carveol improves spatial memory impairment in an amyloidosis rat model.

### 3.3. Effects on the AChE Inhibition

**Figure 3:**
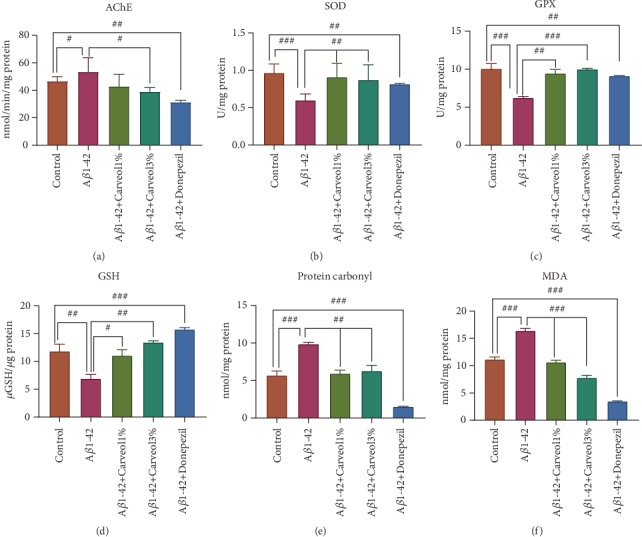
Effects of the inhaled carveol (1% and 3%) on (a) the AChE, (b) SOD, and (c) GPX specific activities and (d) the total content of reduced GSH, (e) protein carbonyl, and (f) MDA levels estimated in the rat hippocampal homogenates of the A*β*1-42-treated rats. Values are the means ± S.E.M. (*n* = 5 animals per group). For Tukey's *post hoc* analyses: (a) control vs. A*β*1-42—^#^*p* < 0.01, control vs. A*β*1-42+Donepezil—^##^*p* < 0.001, and A*β*1-42 vs. A*β*1-42+Carveol1%—^#^*p* < 0.01; (b) control vs. A*β*1-42—^###^*p* < 0.0001, control vs. A*β*1-42+Donepezil—^##^*p* < 0.001, A*β*1-42 vs. A*β*1-42+Carveol1%—^##^*p* < 0.001, and A*β*1-42 vs. A*β*1-42+Carveol3%—^##^*p* < 0.001; (c) control vs. A*β*1-42—^###^*p* < 0.0001, control vs. A*β*1-42+Donepezil—^##^*p* < 0.001, A*β*1-42 vs. A*β*1-42+Carveol1%—^##^*p* < 0.001, and A*β*1-42 vs. A*β*1-42+Carveol3%—^###^*p* < 0.0001; (d) control vs. A*β*1-42—^##^*p* < 0.001, control vs. A*β*1-42+Donepezil—^###^*p* < 0.0001, A*β*1-42 vs. A*β*1-42+Carveol1%—^#^*p* < 0.01, and A*β*1-42 vs. A*β*1-42+Carveol3%—^##^*p* < 0.001; (e) control vs. A*β*1-42—^###^*p* < 0.0001, control vs. A*β*1-42+Donepezil—^###^*p* < 0.0001, A*β*1-42 vs. A*β*1-42+Carveol1%—^#^*p* < 0.01, and A*β*1-42 vs. A*β*1-42+Carveol3%—^##^*p* < 0.001; (f) control vs. A*β*1-42—^###^*p* < 0.0001, control vs. A*β*1-42+Donepezil—^###^*p* < 0.0001, A*β*1-42 vs. A*β*1-42+Carveol1%—^###^*p* < 0.0001, and A*β*1-42 vs. A*β*1-42+Carveol3%—^###^*p* < 0.0001.

The modulatory activity of (-)-*cis*-carveol on AChE activity was also determined. AChE exhibited a secondary noncholinergic function, including the processing and deposition of A*β*. Currently, the therapeutic option for AD patients is the use of AChE inhibitors, which gives only a symptomatic relief [44]. Compared with the control group, A*β*1-42 injection promoted increasing of the hippocampal AChE activity (*p* < 0.01) (Figure 3). On the contrary, (-)-*cis*-carveol (1% and 3%), but especially the dose of 3%, significantly decreased the AChE activity in the rat hippocampus (*p* < 0.01 for (-)-*cis*-carveol 3%) as compared to A*β*1-42 rats.

Accumulating evidence has reported an AChE inhibitory activity of carvacrol. Seo et al. [45] noticed that among the identified constituents, carvacrol inhibited the AChE activity with an IC_50_ value of 0.057 mg/mL. In another study, López et al. [46] indicated that carvone was able to inhibit the AChE activity. Postu et al. [26] demonstrated that *Pinus halepensis* essential oil improved memory deficits determined by A*β*1-42 injection by modulating the AChE action. Thus, (-)-*cis*-carveol should be considered an anti-AChE agent to attenuate cholinergic deficits produced after A*β*1-42 injection. Therefore, this may be one of the mechanisms of (-)-*cis*-carveol improving memory performance in the Y-maze and radial arm maze tests.

### 3.4. Effects on the Oxidative Stress Markers in the Hippocampal Homogenates

A*β*-associated oxidative stress is known to play an essential role in the etiology and the pathogenesis of AD [47]. The A*β*1-42 group had a significant decrease of SOD- (*p* < 0.01) and GPX- (*p* < 0.01) specific activities along with a significant decrease of the total content of reduced GSH (*p* < 0.001) and significantly higher levels of protein carbonyl (*p* < 0.001) and MDA (*p* < 0.001) as a valid biomarker of lipid peroxidation when compared to the control group. Additionally, treatment of the A*β*1-42 group with (-)-*cis*-carveol (1% and 3%) significantly attenuated these alterations as compared to A*β*1-42-treated rats.

Supporting data suggested that the downregulation of the antioxidants determines neurodegeneration [48]. Thus, the downregulation of SOD contributed to A*β* oligomerization and initiated cognitive impairment [49]. Also, GSH is reported to have an essential role in the brain detoxification of reactive oxygen species [50]. MDA is a crucial indicator of lipid peroxidation. Moreover, it was reported that A*β*1-42 induced an overt enhancement of oxidative stress characterized by an increase in MDA level [51]. In the present work, (-)-*cis*-carveol demonstrated antioxidant activity, as evidenced by enhanced activity of SOD and GPX, increased GSH level and reduced protein carbonyl and MDA level in the brain of AD rats.

Several studies demonstrated the antioxidant activities of single or multiple monoterpenes [52, 53]. Postu et al. [26] showed antioxidant activity of the *Pinus halepensis* essential oil mainly attributed to its phytoconstituents. Kaur et al. [54] demonstrated that among all the tested compounds from *Anethum graveolens* L. essential oil for their antioxidant activity, (-)-*cis*-carveol and perillyl alcohol were most productive (IC_50_ values < 0.16 mg/mL). Also, in a recent study, Ibrahim et al. [55] demonstrated antioxidant activity of the volatile components of the peppermint essential oil, including (-)-*cis*-carveol. Therefore, these results support the idea that (-)-*cis*-carveol is an effective antioxidant for AD prevention or treatment.

Regarding the limitations of our study, the levels of the (-)-*cis*-carveol in the blood-mediated observed effects in the experimental groups were still unknown. Furthermore, Jäger et al. [56] demonstrated that R-(-)- and S-(­)-carvone rapidly penetrated the skin of healthy subjects leading to significantly different blood levels. The highest concentration was found in the first 4 hours following exposure. Therefore, we presumed that (-)-*cis*-carveol exhibited the positive effects in mediating the improvement of the memory processes via decreasing brain oxidative stress and inhibiting the hippocampal AChE activity in the Sco-treated rats.

## 4. Conclusions

In summary, the present study demonstrated that (-)-*cis*-carveol was able to reverse the cognitive deficits resulting from the A*β*1-42 treatment. Additionally, we also established that the positive effects of (-)-*cis*-carveol could be mediated by decreasing brain oxidative stress and regulation of AChE activity. Therefore, the present work suggested that (-)-*cis*-carveol provides neuroprotection against A*β*1-42 and can be regarded an alternative therapeutic agent for dementia-related neurological conditions, including AD.

## Data Availability

The data used to support the findings of this study have been deposited in the website of the Federal University of Sergipe at https://http://www.sigaa.ufs.br/sigaa/public/programa/defesas.jsf?lc=pt_BR&id=719 and published in file:///C:/Users/damia/Downloads/molecules-20-19649.pdf.
